# Stroma AReactive Invasion Front Areas (SARIFA) improves prognostic risk stratification of perioperative chemotherapy treated oesophagogastric cancer patients from the MAGIC and the ST03 trial

**DOI:** 10.1038/s41416-023-02515-4

**Published:** 2023-12-20

**Authors:** Bianca Grosser, Jake Emmerson, Nic G. Reitsam, David Cunningham, Matthew Nankivell, Ruth E. Langley, William H. Allum, Martin Trepel, Bruno Märkl, Heike I. Grabsch

**Affiliations:** 1 Pathology, Medical Faculty Augsburg, University of Augsburg, Augsburg, Germany; 2 Leeds Institute of Clinical Trials Research, University of Leeds, Leeds, UK; 3 Department of Medicine, Royal Marsden Hospital, Sutton, Surrey, UK; 4 Medical Research Council Clinical Trials Unit at University College London, London, UK; 5 Department of Oncology and Department of Surgery, Royal Marsden NHS Foundation Trust, London, UK; 6 Haematology and Oncology, Medical Faculty Augsburg, University of Augsburg, Augsburg, Germany; 7 Department of Pathology, GROW School for Oncology and Reproduction, Maastricht University Medical Center+, Maastricht, The Netherlands; 8 Division of Pathology and Data Analytics, Leeds Institute of Medical Research at St James’s University, University of Leeds, Leeds, UK

**Keywords:** Gastric cancer, Prognostic markers

## Abstract

**Background:**

Tumour-associated fat cells without desmoplastic stroma reaction at the invasion front (Stroma AReactive Invasion Front Areas (SARIFA)) is a prognostic biomarker in gastric and colon cancer. The clinical utility of the SARIFA status in oesophagogastric cancer patients treated with perioperative chemotherapy is currently unknown.

**Methods:**

The SARIFA status was determined in tissue sections from patients recruited into the MAGIC (*n* = 292) or ST03 (*n* = 693) trials treated with surgery alone (S, MAGIC) or perioperative chemotherapy (MAGIC, ST03). The relationship between SARIFA status, clinicopathological factors, overall survival (OS) and treatment was analysed.

**Results:**

The SARIFA status was positive in 42% MAGIC trial S patients, 28% MAGIC and 48% ST03 patients after pre-operative chemotherapy. SARIFA status was related to OS in MAGIC trial S patients and was an independent prognostic biomarker in ST03 trial patients (HR 1.974, 95% CI 1.555–2.507, *p* < 0.001). ST03 patients with lymph node metastasis (ypN + ) and SARIFA-positive tumours had poorer OS than patients with ypN+ and SARIFA-negative tumours (*p*_logrank_ < 0.001).

**Conclusions:**

The SARIFA status has clinical utility as prognostic biomarker in oesophagogastric cancer patients irrespective of treatment modality. Whilst underlying biological mechanisms warrant further investigation, the SARIFA status might be used to identify new drug targets, potentially enabling repurposing of existing drugs targeting lipid metabolism.

## Introduction

Gastric cancer is ranked as the fifth most common cancer worldwide accounting for ~769,000 cancer-associated deaths in 2020 [1]. The introduction of perioperative or neoadjuvant combination chemotherapy significantly improved the outcome in patients with tumour-node-metastasis (TNM) stage II or III gastric or oesophagogastric cancers [2]. The greatest benefit from perioperative combination chemotherapy seems to come from the preoperative part as in most trials, including MAGIC and ST03, a significant number of patients did not complete the postoperative treatment as originally planned in the protocol. Despite this progress, death due to locally recurrent disease or distant metastasis remains a major challenge [3]. In everyday clinical practice, the clinical decision on the postoperative treatment and surveillance strategy is highly relevant with regard to tolerability and quality of life. Therefore, there remains an urgent clinical need to identify a biomarker which can predict the risk of recurrent disease and/or overall survival (OS) after neoadjuvant therapy and surgical resection in order to personalise postoperative follow-up and treatment.

Histomorphological biomarker such as tumour budding [4] or tumour-stroma ratio [5], as well as a several molecular classifications have been proposed to predict prognosis or response to therapy in oesophagogastric cancer patients [6, 7]. However, to date, none of these has been introduced into routine clinical practice and TNM disease stage continues to be the only clinically used prognostic parameter informing treatment decision in oesophagogastric cancer patients.

**Fig. 1:**
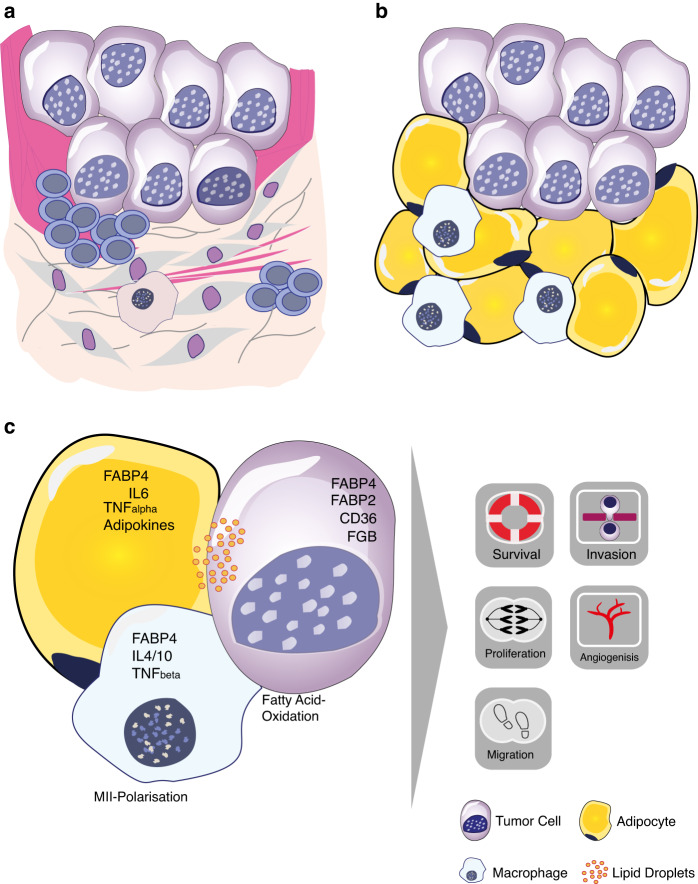
Proposed biological mechanisms at Stroma AReactive Invasion Front Areas (SARIFA). **a** Usually, tumour cell invasion induces a desmoplastic stroma reaction at the invasive edge. Inflammatory cells (mostly lymphocytes) are seen close to tumour cells in the stroma. Cancer-associated fibroblasts proliferate, producing extracellular matrix such as collagen, whereby the stroma becomes more dense and less cellular over time. **b** In SARIFA-positive tumours, there is at least focally no desmoplastic reaction at all. The host’s adipocytes are located directly adjacent to the tumour cells and a direct interaction between tumour cells and adipocytes seems possible to the advantage of the tumour. **c** Crosstalk between tumour cells, adipocytes and macrophages may induce a change in the metabolism of the tumour cells resulting in several tumour promoting effects. Tumour-associated fat cells may serve as an exogenous source of fatty acids. Fatty acid binding proteins (FABP) seem to be of particular importance with a supposed function for the intracellular lipid transport to specific compartments [20–22]. Our own findings suggest that FABP4 is upregulated in tumour cells of SARIFA-positive gastric cancers with concomitant increased CD36 (fatty acid translocase) protein expression. Adipocytes promote MII polarisation of macrophages which may lead to immune suppression [23]. The crosstalk may therefore promote tumour cell survival, proliferation, migration, invasion, and angiogenesis.

We previously identified tumour-associated fat cells without desmoplastic stroma reaction at the invasion front (Stroma Areactive Invasion Front Areas (SARIFA)) as a new histomorphological prognostic biomarker in patients with gastric or colon cancer from a single hospital [8, 9]. We demonstrated that the SARIFA status (positive vs negative) can be determined on routine Haematoxylin & Eosin (HE) stained tissue sections with high interobserver agreement. Moreover, we provided the first evidence for an interaction between adipocytes and tumour cells using digital spatial profiling suggesting that SARIFA-positive tumours may have an altered immune response (Fig. 1).

Based on these initial observations, we hypothesised that patients with SARIFA-positive oesophagogastric cancer have a poor prognosis irrespective whether they have been treated with surgery alone or perioperative combination chemotherapy.

The aim of the study was to establish whether the SARIFA status has clinical utility as a predictive and/or prognostic biomarker in oesophagogastric cancer patients with locally advanced resectable disease. Therefore, we determined the SARIFA status in HE stained tissue sections from resection specimens of 985 patients recruited into the United Kingdom Medical Research Council (MRC) Adjuvant Gastric Infusional Chemotherapy (MAGIC) and UK MRC ST03 randomised trials and investigated the relationship between SARIFA status, clinicopathological variables, OS and treatment.

## Material and methods

### MAGIC and ST03 trial patients

The MAGIC trial was an open-label, multicenter, phase 3 clinical trial randomising patients with resectable oesophagogastric adenocarcinoma to either perioperative epirubicin, cisplatin, and 5-fluorouracil chemotherapy plus surgery (ECF) or surgery alone (S) between July 1994 and April 2002. Of the 503 randomised patients, 453 (90%) patients proceeded to surgical resection. For a previous study, a single representative Haematoxylin/Eosin (HE) stained tissue section with tumour was collected and scanned at 40x magnification (Leica Aperio AT scanner, University of Leeds, Leeds, UK). Slides from 335 resection specimens were available for review for the purpose of the current study. Patients with complete pathological response were not included in the current study.

The UK MRC ST03 clinical trial was an open-label, multicenter, phase 3 clinical trial randomising 1063 patients with resectable gastroesophageal adenocarcinoma to either perioperative epirubicin, cisplatin, and capecitabine (ECX) chemotherapy or ECX plus Bevacizumab (ECX-Bev) between October 2007 and March 2015 [10]. Of these, 903 (85%) patients proceeded to surgical resection. During the trial, 20,277 slides from 799 resection specimens were collected prospectively and scanned at 40x magnification (Leica Aperio AT scanner, University of Leeds, Leeds, UK). Slides from ST03 patients with complete pathological response (*n* = 56) or those taking part in the Lapatinib feasibility substudy (*n* = 6, 0.8%) were excluded from analyses. HE stained tissue sections from pretreatment (diagnostic) endoscopic biopsies of 100 randomly selected patients were also included in this study.

Individual patient clinicopathological characteristics (sex, age, body mass index (BMI), depth of invasion (y)pT, lymph node status (y)pN, primary tumour regression grade, tumour localisation and resection margin status), and outcome data were retrieved from MRC Clinical Trial Unit London (UK) databases, original histopathology reports or established during review of scanned slides.

This study was approved by the UK NRES Committee London-South East (IRAS ID 144220).

### SARIFA status assessment

The SARIFA status was determined reviewing all available digital HE stained resection specimen sections from both trials². In the MAGIC trial, a single representative tumour section was available per patient only, whereas in the ST03 trial, multiple slides with primary tumour were available from most of the resection specimen. SARIFA positivity was defined as presence of an area within the primary tumour where at least a single tumour gland or a group of at least five tumour cells are located directly adjacent to adipocytes. In the normal wall of the stomach or oesophagus, adipocytes are most commonly seen in the subserosa, but can also be present in the submucosa or around large vessels within the wall. Presence of one area classified as SARIFA-positive was sufficient to classify a whole tumour as SARIFA-positive, size of the area was not considered. If SARIFA-positive areas were only observed in one of the tumour containing slides in the ST03 trial, the number of tumour cells, SARIFA areas and adipocytes involved in the SARIFA area were noted. If there was no SARIFA-positive area in any of the slides, the whole tumour was classified as SARIFA-negative. Cases with complete pathological regression (e.g. no tumour on any of the slides), intramucosal cancer only, poor slide quality or other technical problems were classified as non-assessable. The assessment rules were the same for all resection slides irrespective of the type of treatment.

In addition, 100 pretreatment endoscopic biopsies from ST03 patients were screened for the presence of fat to assess feasibility of determining SARIFA status in pretreatment biopsies.

All slides were assessed by one observer (BG) blinded to any clinicopathological information. A second observer (HG) reviewed all the cases that were deemed as not-assessable by the first observer (MAGIC: *n* = 21 (6%), ST03: *n* = 44 (6%)).

### Statistical analyses

The relationship between the SARIFA status and clinicopathological characteristics (primary tumour regression grade according to Mandard [11], sex, depth of invasion ((y)pT), lymph node status ((y)pN), resection margin (R) status, treatment, tumour location, BMI) was analysed using the Kruskal-Wallis test, Mann-Whitney U test or Chi square test as appropriate. Analyses were stratified by treatment arm in the MAGIC trial as we were interested in comparing the relationship between the SARIFA status and survival in surgery alone treated patient with that of patients treated with perioperative chemotherapy. As patients in both treatment arms in ST03 received perioperative chemotherapy and the initial analysis showed that the relationship between the SARIFA status and survival was similar in both treatment arms, we did not stratify the analyses by treatment in ST03. In the ST03 trial, we additionally classified patients combining the SARIFA status and the lymph node status into (a) SARIFA-positive + ypN0, (b) SARIFA-positive + ypN+ (N+: presence of lymph node metastasis irrespective of number of metastases), (c) SARIFA-negative + ypN0 and (d) SARIFA-negative + ypN+ to analyse the relationship of this combined classifier with OS.

The primary outcome measure was OS from the date of randomisation with surviving patients censored at their date of last follow-up. For univariable survival analyses,

Kaplan–Meier estimates were compared using log-rank tests, and hazard ratio (HR)s were calculated from Cox proportional hazard models. Multivariable analysis was performed using a Cox proportional hazard model including known prognostic factors ypT, ypN, ypR, and Mandard TRG.

Statistical analyses were performed using SPSS (Version 28, IBM Corp., Armonk, NY, USA) and R Version 4.2.1.

## Results

**Fig. 2:**
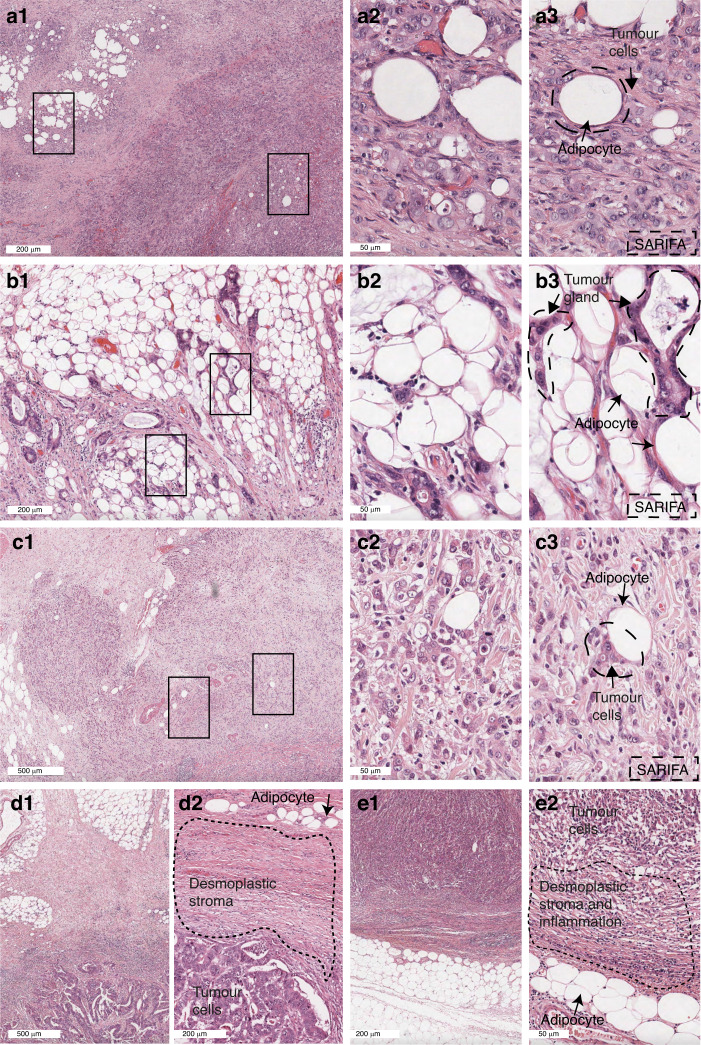
Haematoxylin & Eosin-stained images of SARIFA-positive and SARIFA-negative gastric cancer. **a** SARIFA-positive cancer from a surgery alone treated MAGIC trial patient showing tumour cells directly adjacent to adipocytes without desmoplastic stroma reaction (scale bar 200 µm; inserts 50 µm); a1: overview, a2 inset 1: showing several large fat cells (optically empty spaces) directly adjacent to tumour cells. a3 inset 2: tumour cells and fat cells annotated. **b** SARIFA-positive cancer from a ST03 trial patient after neoadjuvant chemotherapy showing extensive fat in the subserosa, no desmoplastic stroma reaction (scale bar 200 µm; inserts 50 µm). b1: overview, b2 inset 1: showing several large fat cells (optically empty spaces) directly adjacent to tumour cells. b3 inset 2: tumour cells and fat cells annotated. **c** SARIFA-positive cancer from a ST03 trial patient after neoadjuvant chemotherapy showing fat adjacent to tumour cells in the submucosa, no desmoplastic stroma reaction (scale bar 500 µm; inserts 50 µm). c1: overview, c2 inset 1: showing several large fat cells (optically empty spaces) directly adjacent to tumour cells. c3 inset 2: with annotated tumour cells and fat cells. **d** SARIFA-negative cancer from a surgery alone treated MAGIC trial patient showing a desmoplastic stroma reaction between tumour and fat at the invasive front (scale bar 500 µm; insert 200 µm). d1: overview, d2: tumour cells, fat cells, and desmoplastic stroma annotated. **e** SARIFA-negative cancer from a ST03 trial patient after neoadjuvant chemotherapy showing desmoplastic stroma with inflammation between tumour and fat at the invasive front (scale bar 200 µm; insert 50 µm). e1: overview, e2: tumour cells, fat cells and desmoplastic stroma with inflammation annotated.

SARIFA status was evaluated in primary tumour slides from 335 patients in the MAGIC trial and 799 patients in the ST03 trial. Example images for SARIFA-positive and SARIFA-negative areas are presented in Fig. 2.

The SARIFA status was classified as non-assessable in 43 (12.8%) and 106 (13.3%) tumours in the MAGIC trial and ST03 trial, respectively, leaving 292 patients from the MAGIC trial (158 (54.1%) S patients, 134 (45.9%) ECF patients) and 693 patients from the ST03 trial (364 (52.5%) ECX patients, 329 (47.5%) ECX +Bev patients) for final analyses, see Supplementary Fig. S1.

In the MAGIC trial, 103 (35.3%) tumours were classified SARIFA-positive. The frequency of SARIFA-positive tumours was significantly higher in S patients compared to ECF patients (S: *n* = 66, 41.8%, ECF: *n* = 37, 27.6%, *p* = 0.012). Therefore, all subsequent analyses in the MAGIC trial were stratified by treatment arm.

In the ST03 trial, 336 (48.5%) tumours were classified SARIFA-positive (ECX: *n* = 172 (51.2%), ECX + Bev: *n* = 164 (48.8%)), and 357 (48.5%) were classified as SARIFA-negative (ECX: *n* = 192 (53.8%), ECX + Bev: *n* = 165 (46.2%)). There was no significant difference in the frequency of SARIFA positivity or negativity between treatment arms in the ST03 trial (*p* = 0.458).

A single SARIFA-positive area was observed in a small proportion of cases. In the ST03 trial, 69 (21%) of 336 SARIFA-positive tumours had a single SARIFA-positive area although they had several tumour containing slides. Of these, one case had five tumour cells next to a fat cell and three cases showed a single tumour gland adjacent to a fat cell.

### Relationship between SARIFA status and clinicopathologic characteristics

Clinicopathological characteristics at baseline for both trials stratified by SARIFA status and treatment arm, are provided in Tables 1 and 2.

**Table 1:** Baseline characteristics of MAGIC trial patients stratified by treatment arm and SARIFA status.

		MAGIC trial—perioperative ECF patients							MAGIC trial—surgery alone patients						
		All patients *n* = 134		SARIFA-negative *n* = 97 (72.4%)		SARIFA-positive *n* = 37 (27.6%)		*p*-value	All patients *n* = 158		SARIFA-negative *n* = 92 (58.2%)		SARIFA-positive *n* = 66 (41.8%)		*p-*value
Median age (range) [years]		63.5 (35.0–79.0)		64.0 (35.0–79.0)		62.0 (36.0–75.0)		0.388	62.0 (23.0–81.0)		61.0 (23.0–81.0)		63.5 (35.0–77.0)		0.498
Median follow-up [years]		4.2 (3.8–4.5)		4.2 (3.7–4.7)		4.0 (1.4–6.6)		0.628	3.9 (3.7–4.1)		4.0 (3.8–4.2)		3.1 (1.3–4.8)		0.984
Median BMI (range) [kg/m2]		25.9 (17.2–37.3)		25.7 (17.2–33.5)		26.6 (19.1–37.3)		0.330	-		-		-		-
		*n*	%	*n*	%	*n*	%		*n*	%	*n*	%	*n*	%	
Sex	female	24	18	16	16	8	22	0.489	43	27	25	27	18	27	0.989
	male	110	82	81	84	29	78		115	73	67	73	48	73	
(y)pT	T1	12	10	11	13	1	3	0.539	10	7	9	11	1	2	**0.033**
	T2	44	37	29	34	15	44		42	29	28	34	14	23	
	T3	57	48	41	48	16	47		86	59	41	49	45	73	
	T4	5	5	4	5	1	3		7	5	5	6	2	2	
(y)pN	N0	37	32	32	39	5	15	**0.013**	42	30	27	33	15	25	0.285
	N1 or more	78	68	50	61	28	85		99	70	54	67	45	75	
Resection margin status	negative	99	88	73	91	26	81	0.274	118	86	73	89	45	82	0.282
	positive	13	12	7	9	6	19		19	14	9	11	10	18	
Tumour location	oesophagus	21	16	21	22	0	0	**0.004**	18	11	11	12	7	11	0.695
	OGJ	15	11	12	12	3	8		18	11	12	13	6	9	
	stomach	98	73	64	66	34	92		122	78	69	75	53	80	
Histological tumour type (Lauren)	intestinal	87	65	68	70	19	51	0.086	94	59	55	60	39	59	0.838
	diffuse	32	24	21	22	11	30		40	25	22	24	18	27	
	mixed	15	11	8	8	7	19		24	16	15	16	9	14	
Mandard primary tumour regression grade	2	23	17	20	21	3	8	**0.045**	-	-	-	-	-	-	-
	3	52	39	31	32	21	57		-	-	-	-	-	-	
	4	41	31	33	34	8	22		-	-	-	-	-	-	
	5	18	13	13	13	5	13		-	-	-	-	-	-	

Numbers do not always add up to 134 or 158, respectively, because of missing values. *P*-values refer to the comparison between SARIFA-positive and SARIFA-negative within the treatment arm.

Bold values indicate statistical significance *p* < 0.05.

*ECF* epirubicin/cisplatin/5-fluorouracil, *BMI* body mass index, *(y)pT* pathological depth of invasion, *(y)pN* pathological lymph node status, *OGJ* oesophagogastric junction.

**Table 2:** Baseline characteristics of ST03 trial patients stratified by treatment arm and SARIFA status.

		ST03—perioperative ECX patients							ST03—perioperative ECX + bevacizumab patients						
		All patients *n* = 364		SARIFA-negative *n* = 192 (53%)		SARIFA-positive *n* = 172 (47%)		*p-*value	All patients *n* = 329		SARIFA-negative *n* = 165 (50%)		SARIFA-positive *n* = 164 (50%)		*p-*value
Median age (range) [years]		63.0 (31.0–79.0)		63.0 (36.0–79.0)		62.5 (31.0–78.0)		0.073	64.0 (28.0–79.0)		64.0 (28.0–79.0)		63.0 (34.0–77.0)		0.375
Median follow-up [years]		5.3 (4.9–5.6)		4.9 (4.4–5.5)		5.5 (4.7–6.2)		0.665	5.2 (4.8–5.6)		5.0 (4.3–5.7)		5.3 (4.9–5.7)		0.042
Median BMI (range) [kg/m2]		26.3 (17.4–76.7)		26.1 (17.4–70.8)		26.9 (19.0–76.7)		0.089	26.2 (17.6–62.7)		26.2 (18.4–42.5)		26.3 (17.6–62.7)		0.627
		*n*	%	*n*	%	*n*	%		*n*	%	*n*	%	*n*	%	
Sex	female	79	22	38	20	41	24	0.350	63	19	34	21	29	18	0.500
	male	285	78	154	80	131	76		266	81	131	79	135	82	
ypT	T1	27	7	23	12	4	2	**<0.001**	35	11	32	20	3	2	<**0.001**
	T2	146	40	94	49	52	30		124	38	76	46	48	29	
	T3	151	42	63	33	88	51		146	44	54	33	92	56	
	T4	38	11	10	6	28	17		23	7	2	1	21	13	
ypN	N0	135	37	92	48	43	25	**<0.001**	114	35	86	52	28	17	**<0.001**
	N1/2/3	227	63	98	52	129	75		215	65	79	48	136	83	
Resetion margin status	negative	260	72	157	83	103	60	**<0.001**	237	72	137	84	100	61	<**0.001**
	positive	100	28	32	17	68	40		90	28	27	16	63	39	
Histological tumour type	intestinal	259	71	149	78	110	64	**0.004**	234	71	131	79	103	63	**0.001**
	diffuse	105	29	43	22	62	36		95	29	34	21	61	37	
Tumour location	oesophagus	57	16	32	17	25	15	0.855	44	13	25	15	19	12	0.603
	OGJ	117	32	61	32	56	33		91	28	46	28	45	27	
	stomach	190	52	99	51	91	52		194	59	94	57	100	61	
Mandard primary tumour regression grade	2	12	3	12	6	0	0	**<0.001**	16	5	15	9	1	1	**<0.001**
	3	86	24	64	34	22	13		83	25	61	37	22	13	
	4	220	61	95	50	125	73		181	56	72	44	109	67	
	5	45	12	20	10	25	14		46	14	15	10	31	19	

Numbers do not always add up to 364 or 329, respectively, because of missing values. P-values refer to the comparison between SARIFA-positive and SARIFA-negative within the treatment arm.

Bold values indicate statistical significance *p* < 0.05.

*ECX* epirubicin/cisplatin/capecitabine, *BMI* body mass index, *(y)pT* pathological depth of invasion, *(y)pN* pathological lymph node status, *OGJ* oesophagogastric junction.

In MAGIC ECF patients, tumours located in the stomach were more likely to be SARIFA-positive compared to tumours in the lower oesophagus or oesophagogastric junction (*p* = 0.004). Patients with SARIFA-positive tumours were more likely to have regional lymph node metastasis (*p* = 0.013). In MAGIC S patients, SARIFA positivity was associated with greater depth of invasion (pT) (*p* = 0.033). No relationship was seen between SARIFA status and other clinicopathological characteristics, see Table 1.

In the ST03 trial, SARIFA-positive tumours showed less primary tumour regression, were associated with higher ypT, presence of lymph node metastases, and positive resection margins in both treatment arms (see Table 2, all *p* < 0.001). SARIFA-positive tumours showed more often diffuse-type histology (ECX: *p* = 0.004; ECX + Bev: *p* < 0.001). No relationship was seen between the SARIFA status and other clinicopathological characteristics, see Table 2.

### SARIFA status and body mass index

BMI data were available for all ST03 trial patients, but only available for 121 patients from the ECF arm in the MAGIC trial. SARIFA status was not associated with BMI in neither of the trials (ST03 *p* = 0.086, MAGIC *p* = 0.330). In both trials, there was no association of SARIFA status with WHO performance status (MAGIC: *p* = 0.472; ST03: *p* = 0.954).

### Relationship between the SARIFA status and overall survival in the MAGIC trial patients

**Fig. 3:**
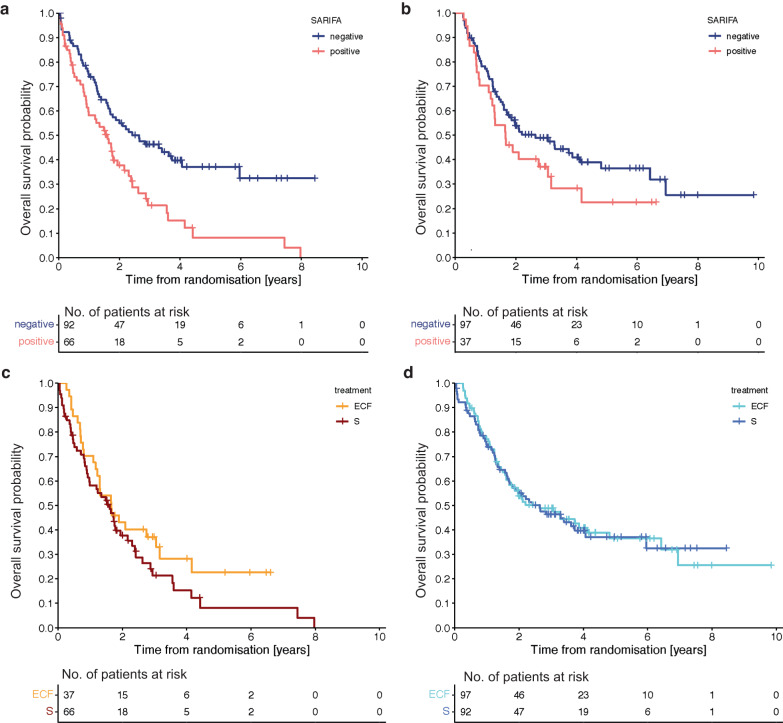
Overall survival analyses in the MAGIC trial patients stratified by treatment arm and SARIFA status. **a** Kaplan Meier analysis of the MAGIC trial patients treated by surgery alone shows that patient with SARIFA-negative tumour have a significantly better survival (HR 1.899; 95 CI (1.285–2.806); *p*_logrank_ = 0.001). **b** Kaplan Meier analysis of the MAGIC trial patients treated with peri-operative chemotherapy seems to suggest improved survival in patients with SARIFA-negative tumours. However, the difference is statistically not significant (HR 1.400; 95 CI (0.878–2.232); *p*_logrank_ 0.155). **c** Kaplan Meier analysis comparing survival in the MAGIC trial patients with SARIFA-positive tumours between treatment arms shows a slightly improved survival after 2 years in the peri-operative chemotherapy arm. However, the difference is statistically not significant (HR 0.728; 95 CI (0.856–2.219); p_logrank_ 0.190). **d** Kaplan Meier analysis comparing survival in the MAGIC trial patients with SARIFA-negative tumours between treatment arms shows no difference in survival (HR 0.985; 95 CI (0.697–1.478); *p*_logrank_ 0.940).

S patients with SARIFA-positive tumours had significantly shorter OS (hazard ratio (HR) 1.899; 95% confidence interval (CI) (1.285–2.806); *p*_logrank_ = 0.001, Fig. 3a). The estimated median (95% CI) OS of S patients with SARIFA-positive tumours was 1.61 years (1.09–2.13 years) compared to 2.65 years (1.34–3.95 years) for S patients with SARIFA-negative tumours. 79% of S patients with SARIFA-positive tumours were dead at the end of the study period compared to 57% of the S patients with SARIFA-negative tumours.

ECF patients with SARIFA-negative tumours seem to have a better survival compared to ECF patients with SARIFA-positive tumours, however, the survival difference was not significant (HR 1.400; 95% CI (0.878–2.232), *p*_logrank_ = 0.155, Fig. 3b). ECF patients with SARIFA-positive tumours had an estimated median (95% CI) OS of 1.65 years (0.95–2.36 years) compared to 2.64 years (1.40–3.89 years) in ECF patients with SARIFA-negative tumours. 30% of the ECF patients with SARIFA-positive tumours were alive at the end of the study period compared to 41% of the ECF patients with SARIFA-negative tumours. Survival of patients with SARIFA-positive tumours treated with perioperative chemotherapy (*n* = 37) seems to be slightly better after 2 years compared to patients with SARIFA-positive tumours treated with surgery only (*n* = 66), however, this difference was not significant (HR 0.728; 95 CI (0.856–2.219); *p*_logrank_ 0.190; Fig. 3c). Patients with SARIFA-negative tumours had similar survival irrespective of treatment modality (HR 0.985; 95 CI (0.697–1.478); *p*_logrank_ 0.940; Fig. 3d).

### Relationship between the SARIFA status and overall survival in the ST03 trial patients

**Fig. 4:**
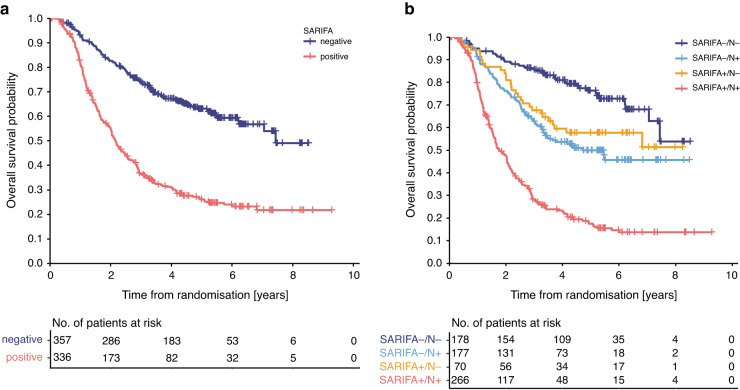
Overall survival analyses in the ST03 trial patients stratified by SARIFA status. **a** Kaplan Meier analysis of all ST03 trial patients shows that patients with SARIFA-negative tumours have a significantly better survival (HR 2.910; 95 CI (2.344–3.613); *p*_logrank_ < 0.001). **b** Kaplan Meier analysis of all ST03 trial patients combining lymph node status (ypN) and SARIFA status shows that patients with SARIFA-positive ypN+ tumours have the poorest survival (HR 6.024, 95% CI 4.364–8.315), followed by SARIFA-negative ypN+ tumours (HR 2.354 95% CI 1.646–3.366), SARIFA-positive ypN0 tumours (HR 1.824 95% CI 1.146–2.904) and patients with SARIFA-negative ypN0 tumours have the best survival (*p*_logrank_ < 0.001).

The initial exploration of the relationship between the SARIFA status and survival per treatment arm showed similar results in both treatment arms (see Supplementary Fig. S2). We therefore decided to analyse patients from both treatment arms together. ST03 patients with SARIFA-positive tumours had significantly shorter OS (HR 2.910; 95% CI (2.344–3.613); *p*_logrank_ < 0.001, Fig. 4a). The estimated median (95% CI) OS of ST03 patients with SARIFA-positive tumours was 2.166 years (1.922–2.410 years) compared to 7.441 years (7.055- (median not reached) years) in patients with SARIFA-negative tumours. 64% of the patients with SARIFA-negative tumours were alive at the end of the study period compared to 30% of the patients with SARIFA-positive tumours.

In multivariable OS analyses including the known prognostic factors ypT, ypN, ypR, and Mandard TRG in the model, the SARIFA status proved to be an independent prognostic factor (HR 1.974, 95% CI 1.555–2.507, *p* < 0.001, Supplementary Table S1).

### Relationship between the SARIFA status, lymph node status and survival in the ST03 trial patients

Combining the SARIFA status and lymph node status (ypN0 vs ypN1 or higher (ypN+)) identified four groups of patients: SARIFA-negative ypN0 (*n* = 178, 26%), SARIFA-negative ypN+ (*n* = 177, 26%), SARIFA-positive ypN0 (*n* = 70, 10%) and SARIFA-positive ypN+ (*n* = 266, 38%). Patients with SARIFA-positive ypN+ tumours had the poorest survival (HR 6.024, 95% CI 4.364–8.315), followed by SARIFA-negative ypN+ tumours (HR 2.354 95% CI 1.646–3.366), SARIFA-positive ypN0 tumours (HR 1.824 95% CI 1.146–2.904) and patients with SARIFA-negative ypN0 tumours having the best survival (reference, *p* < 0.001, Fig. 4b).

## Discussion

The introduction of perioperative or neoadjuvant combination chemotherapy has improved the outcome in patients with locally advanced resectable oesophagogastric cancers. Despite this progress, death due to locally recurrent disease or distant metastasis remains a major challenge in patients with oesophagogastric cancer [3]. In everyday clinical practice, the clinical decision on the postoperative treatment and surveillance strategy is highly relevant with regard to tolerability and quality of life. To address the urgent clinical need to improve stratification for risk of recurrent disease and/or prediction of OS after neoadjuvant therapy and surgical resection in order to personalise postoperative patient management, we evaluated the clinical utility of tumour-associated fat cells without desmoplastic stroma reaction at the invasion front (Stroma AReactive Invasion Front Areas (SARIFA)) in 985 oesophagogastric patients from the the MAGIC and the ST03 trial. Our own previous work showed that SARIFA status can be easily and reproducibly evaluated in routine Haematoxylin/Eosin (HE) stained tissue sections from resection specimens and suggested SARIFA status as new post-operative prognostic biomarker in oesophagogastric cancer patients. A summary of the current evidence including our own previous work regarding underlying mechanisms of the potential tumour promoting effect of tumour-associated adipocytes (CAAs) [12, 13] is presented in Fig. 1.

Our current study confirmed the prognostic value of the SARIFA status in surgery alone treated patients from the MAGIC trial. More importantly, this is the first study to suggest that the SARIFA status can also identify patient with a very poor prognosis when assessed in the post-chemotherapy resection specimens from the ST03 trial patients. Whilst we found a strong relationship between the SARIFA status, depth of tumour invasion, lymph node status and primary tumour regression grade, the SARIFA status nevertheless proved to be related to OS independently of known prognostic factors. In contrast to our findings in the ST03 trial, we only saw a non-significant trend of better survival of patients with SARIFA-negative tumours in the chemotherapy treated MAGIC trial patients. This might be related to the overall much lower number of patients with available SARIFA status in the MAGIC trial or could be related to differences in the sample collection of both trials. The tissue collection in the MAGIC trial was done retrospectively requesting a single representative block with highest tumour content per area, whereas tissue samples were collected prospectively in the ST03 trial requesting all slides from the resection specimen for central review. In particular in the post-chemotherapy resection specimen, a single tissue section selected to provide highest tumour content may not automatically include areas with adipocytes. This sampling bias could potentially also explain the lower frequency of SARIFA positivity in the chemotherapy treated patients in the MAGIC trial compared to the ST03 trial patients.

Lymph node status is known to be one of the most important prognostic factors in oesophagogastric cancer patients, more important than primary tumour regression as we showed in a previous study in the Oe02 and MAGIC trial patients. Combining the SARIFA status of the primary tumour with the lymph node status allowed us to identify subgroups of ypN0 patients with different survival. Whereas patients with SARIFA-negative ypN0 tumours had the best survival, the survival of patients with SARIFA-positive ypN0 tumours was significantly poorer and similar to patients with SARIFA-negative ypN+ tumours. This could suggest that the SARIFA status could be in particular clinically useful to identify ypN0 patients who may require adjuvant treatment which is different to the neoadjuvant regimen.

Lim et al. [14] showed that microvessel density increases in tumours where adipocytes are in contact with tumour cells which might be one of the underlying mechanisms for potential chemotherapy efficacy in SARIFA-positive cancers. Although the survival of SARIFA-positive patients was slightly better when treated with peri-operative chemotherapy compared to surgery alone in the MAGIC trial, this finding was not statistically significant and has to be interpreted with caution due to the small number of patients included in this subgroup analysis.

The main motivation for previous studies into tumour-associated fat cells was the observed association between obesity, cancer incidence, progression and therapy resistance. Results from our study show no association between BMI and SARIFA status. A higher BMI is partly associated with abdominal obesity, which is characterised by increased adipose tissue surrounding the intra-abdominal organs. High BMI does not necessarily mean that there is also more fatty tissue within the organ wall as such [15]. However, the supposed basic mechanism of an adipocyte-driven tumour progression in SARIFA-positive cancers may provide the basis for pharmacological intervention targeting the tumour cell metabolism with existing drugs such as Metformin, FABP4-inhibitors [16, 17] or CD36 [18, 19].

Our study has some limitations. This is a post-hoc analysis from a subset of patients from the the MAGIC and the ST03 trial. However, we have confirmed that the subsets are representative of the trial population who had a surgical resection (data not shown). The retrospective tissue collection of only a single tumour block from the MAGIC trial patients may have introduced bias. The SARIFA status can only be assessed in the resection specimen as fat cells are only present in the submucosa and deeper parts of the wall. As neoadjuvant chemotherapy is standard of care in patients with oesophagogastric cancer in the West, it would be clinically desirable to assess the predictive value of the SARIFA status in the pretreatment endoscopic biopsy in order to inform patient management. However, when screening pretreatment endoscopic biopsies from 100 randomly selected ST03 trial patients, only 4% of patients actually had few adipocytes present in the included submucosa, supporting our view that reliable evaluation of the SARIFA status in endoscopic biopsies is not feasible. Thus, further work is required to establish whether there are histological or molecular features measurable at the luminal (endoscopically reachable) tumour surface which are characteristics for the SARIFA status.

## Conclusions

The results from the current study assessing the SARIFA status in resection specimens of oesophagogastric cancer patients after neoadjuvant chemotherapy from two phase III trials suggest that SARIFA status could be a clinically useful biomarker to identify patients with a high risk of recurrent disease after initial neoadjuvant chemotherapy who might benefit from a different therapeutic approach in the adjuvant setting. Evaluation of the SARIFA status can be done in routinely available patient material e.g. does not require extra material or procedures beyond routine histopathology and has a high interobserver agreement. Thus, this biomarker could be implemented into routine practice at relatively low costs and be reported within a clinically acceptable turnaround time. Further studies are warranted to assess the potential predictive value for already existing vasculature or lipid metabolism targeting agents.

### Supplementary information

Supplementary Information

## Data Availability

The datasets generated and/or analysed during the current study are available from the corresponding author upon reasonable request.
