# Supplementary material for: Stroma AReactive Invasion Front Areas (SARIFA) improves prognostic risk stratification of perioperative chemotherapy treated oesophagogastric cancer patients from the MAGIC and the ST03 trial
Source: Br J Cancer. 2023 Dec 20;130(3):457–66. doi: 10.1038/s41416-023-02515-4 (PMC10844337; doi:10.1038/s41416-023-02515-4)
Supplement: Supplementary file 1 — Supplementary Information [file 41416_2023_2515_MOESM1_ESM.docx]

**Supplementary Figures**


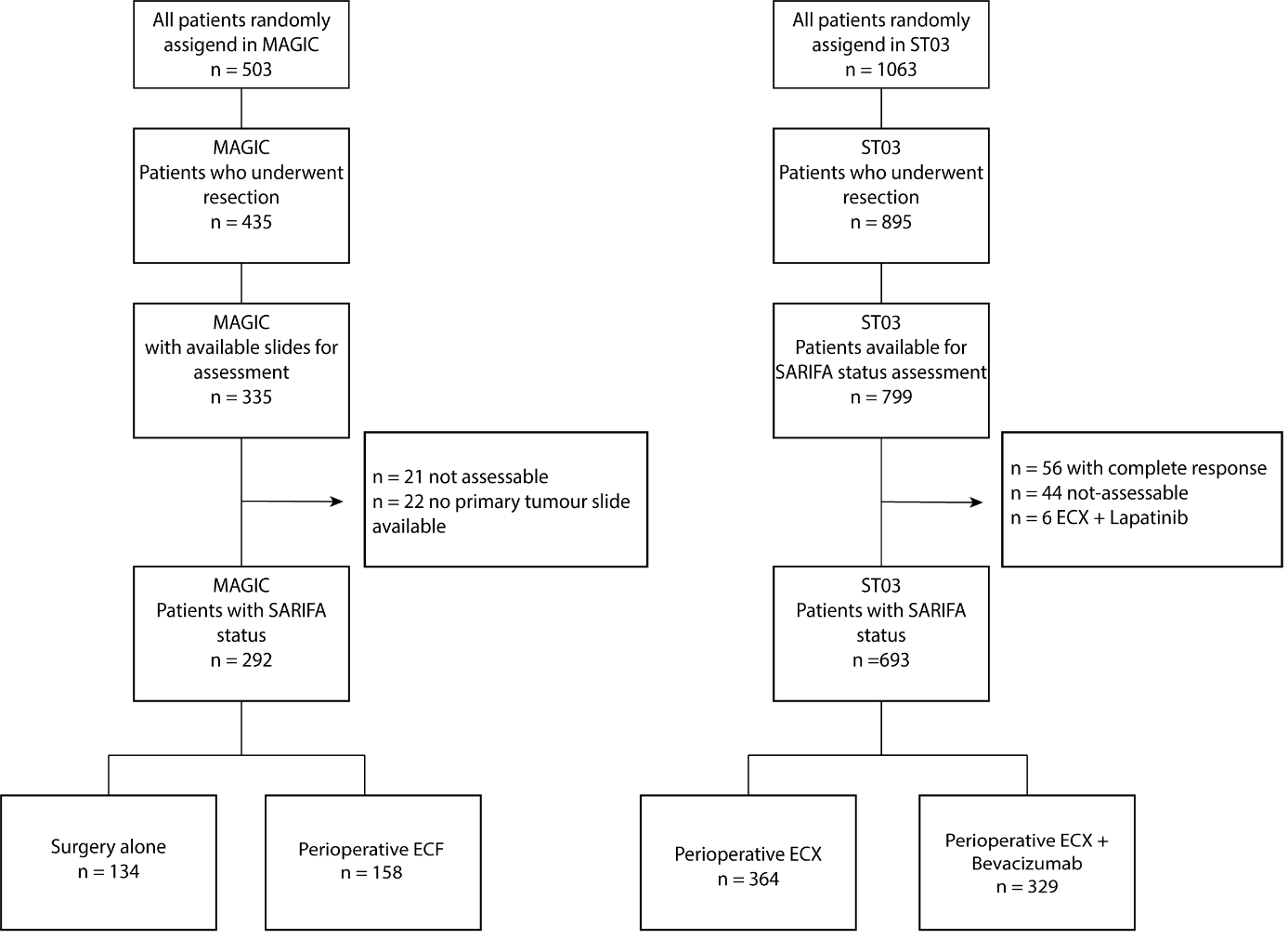


**Supplementary Figure S1:** Consort diagram to illustrate number of patients included in the study from the MAGIC and the ST03 trial. ECF: epirubicin, cis-platin, 5-fluorouracil; ECX: epirubicin, cis-platin, capecitabine

**
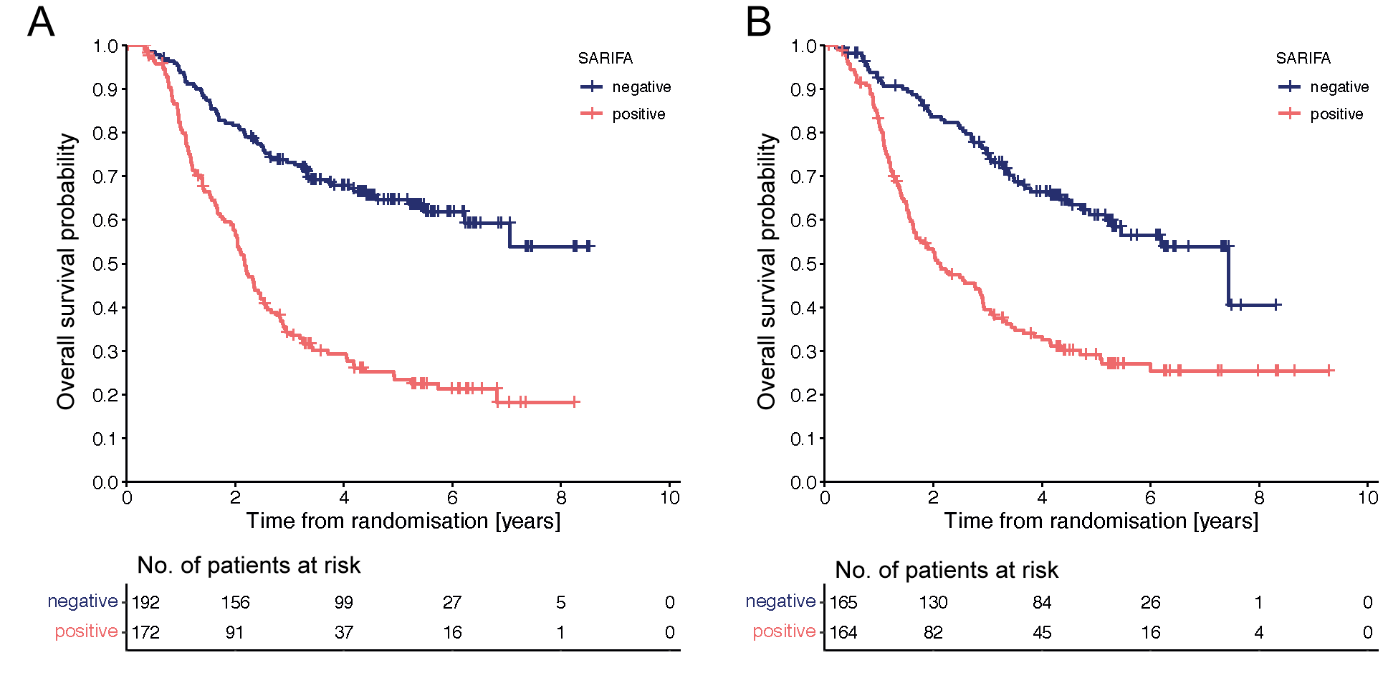
**

**Supplementary Figure S2.** Overall survival analyses in the ST03 trial patients stratified by treatment arm and SARIFA status

**(A)** Kaplan Meier analysis of ST03 patients treated by perioperative ECX shows that patient with SARIFA-negative tumour have a significantly better survival (HR 3.149; 95 CI (2.336 – 4.246); p_logrank_ < 0.001).

**(B)** Kaplan Meier analysis of ST03 patients treated by perioperative ECX + BEV shows that patient with SARIFA-negative tumour have a significantly better survival (HR 2.651 95% CI (1.937 – 3.627); p_logrank_ < 0.001).

**Supplementary Table**

**Supplementary Table S1:** Multivariable overall survival analysis of ST03 trial patients

|  | Overall survival | | | |
| --- | --- | --- | --- | --- |
|  | n | Deaths  n (%) | HR (95% CI) | *p-value* |
| Mandard primary tumour regression | | | | |
| TRG 2 | 28 | 6 (21.4) | 1.000 (ref) |  |
| TRG 3 | 169 | 75 (44.4) | 0.475 (0.201 - 1.122) | 0.090 |
| TRG 4 | 401 | 224 (55.9) | 0.990 (0.689 - 1.424) | 0.958 |
| TRG 5 | 91 | 57 (62.6) | 0.802 (0.598 - 1.076) | 0.142 |
| Depth of invasion | | | | |
| ypT1 | 62 | 10 (16.1) | 1.000 (ref) |  |
| ypT2 | 270 | 118 (43.7) | 0.282 (0.138 - 0.576) | **0.001** |
| ypT3 | 297 | 189 (63.6) | 0.649 (0.453 - 0.931) | **0.019** |
| ypT4 | 61 | 46 (75.4) | 0.748 (0.537 - 1.040) | 0.085 |
| Lymph node status | | | | |
| ypN0 | 249 | 74 (29.7) | 1.000 (ref) |  |
| ypN1/2/3 | 442 | 289 (65.4) | 2.177 (1.655 - 2.864) | **2.729E-8** |
| Resection margin status | | | | |
| negative | 497 | 216 (43.5) | 1.000 (ref) |  |
| positive | 190 | 144 (75.8) | 1.872 (1.493 - 2.349) | **5.715E-8** |
| SARIFA status | | | | |
| negative | 357 | 129 (36.1) | 1.000 (ref) |  |
| positive | 336 | 235 (69.9) | 1.974 (1.555 - 2.507) | **2.341E-8** |

Abbreviations: CI, confidence interval; HR, hazard ratio. TRG: primary tumour regression grade.
ypT: pathological depth of invasion. ypN: pathological lymph node status.
